# Neurocognitive Effects of Cocoa and Red-Berries Consumption in Healthy Adults

**DOI:** 10.3390/nu14010001

**Published:** 2021-12-21

**Authors:** Joaquín García-Cordero, Alicia Pino, Constanza Cuevas, Verónica Puertas-Martín, Ricardo San Román, Sonia de Pascual-Teresa

**Affiliations:** 1 Departamento de Metabolismo y Nutrición, Instituto de Ciencia y Tecnología de Alimentos y Nutrición (ICTAN-CSIC), C/José Antonio Novais, 10, 28040 Madrid, Spain; j.garcia@ictan.csic.es (J.G.-C.); apifer@alumni.uv.es (A.P.); 2 Hospital 12 de Octubre, 28041 Madrid, Spain; constanzaece@gmail.com (C.C.); veronica.hurdes@gmail.com (V.P.-M.); rsan@salud.madrid.org (R.S.R.); 3 Facultad de Educación, Universidad Internacional de la Rioja, 26006 Logrono, Spain

**Keywords:** neurocognition, polyphenols, cocoa, red berries

## Abstract

In recent decades, the elderly population has increased at higher rates than any other population group, resulting in an increase in age-related diseases such as neurodegenerative and cognitive impairment. To address this global health problem, it is necessary to search for new dietary strategies that can prevent the main neurocognitive problems associated with the ageing process. Therefore, the aim of the present study was to analyze the effect of cocoa flavanols and red berry anthocyanins on brain-derived neurotrophic factor (BDNF) and nerve growth factor receptor (NGF-R) and to stablish the possible improvement in cognitive performance by using a battery of neurocognitive tests that included the Verbal Learning Test Spain-Complutense, the Spatial Recall Test 10/36 BRB-N, the Wechsler Adult Intelligence Scale III and IV, the STROOP Task and the Tower of London Test. A randomized, double-blind, parallel-group study was performed in 60 healthy volunteers between 50 and 75 years old who consumed a cocoa powder, a red berries mixture or a combination of both for 12 weeks. After the intervention, we observed a reduction in the time needed to start (*p* = 0.031) and finish (*p* = 0.018) the neurocognitive test known as the Tower of London in all groups, but the decrease in time to finish the task was more pronounced in the intervention with the combination of cocoa-red berries group. We failed to show any significant difference in BDNF and NGF-R sera levels. However we found a negative correlation between BDNF and the number of movements required to finish the TOL in women (*p* = 0.044). In conclusion, our study showed an improvement in executive function, without any change in neurotrofin levels, for all intervention arms.

## 1. Introduction

According to the latest demographical statistics, the older adult population (over 65 years old) is increasing at a much higher rate than other age groups. In 2019, there were 703 million people over the age of 65 around the world, a number that will increase to 1.5 billion by 2050 according to the latest estimates [1] This dramatic growth in this segment of the population is occurring at a higher rate in developed countries. For example, in the European Union at the beginning of 2018 there were more than 101.1 million people over the age of 65 in the 28 member states, corresponding to 19.7% of the total population, but by 2050 this number is expected to increase to 149.2 million inhabitants corresponding to 25.8% of the total population [2]. Although population ageing is the result of an improvement in health systems and overall quality of life [2], it entails a number of complications and challenges for society in the health, economic and social spheres. Ageing is a physiological process involving a decline in the functional capacity of the organism as a result of alterations in multiple molecular pathways [3]. As a result, this population is particularly susceptible to developing a wide range of chronic pathologies, including cognitive impairment and neurodegenerative diseases [3,4].

In 2018, the American Academy of Neurology determined that between 15–20% of people over the age of 65 suffered from mild cognitive impairment (MCI), a condition in which individuals show decay in cognitive function with a minimal deterioration in the instrumental activities of daily living [5]. In the general population, with normal ageing, some neurocognitive changes such as decline in attention (especially with complex tasks), verbal fluency, visual recognition and a slight loss in memory (especially in spontaneous retrieval of information and prospective and source memory) and executive function can be seen [6]. However, in MCI this loss in cognition and memory is more pronounced and may even progress into more malignant forms of neurocognitive decline, such as dementia; a condition that involves the loss of mental functions, so that the affected person becomes completely dependent. Dementia shows a prevalence as high as 10–12% in this same population. This decline in cognitive function can be examined through the use of various neurocognitive tests set up to detect irregularities in mental processes [5], but also through changes in the structure and functioning of certain brain areas by using, for example, magnetic resonance imaging (MRI) to obtain high-resolution images of brain anatomy or functional MRI to detect differences in brain functioning through the oxygen consumption of those tissues [7]. During ageing, a number of changes in brain physiology occur, including loss of white matter volume in the putamen, accumbens nucleus, thalamus, frontal and temporal cortex [8,9]; hippocampal atrophy as a result of reduced synaptic density and neuronal size [6,9], loss of vascular density, increased oxidative stress, failure of certain neurotransmitters such as γ-aminobutyric acid (GABA) [10] and dysregulation of key proteins in regions involved in learning and memory such as the α-amino-3-hydroxy-5-methyl-4-isoxazolepropionic acid (AMPA) and N-methyl-D-aspartate (NMDA) glutamate receptors [8]. Additionally, multiple studies have shown that ageing involves a decrease in the levels of neurotrophic factors such as brain-derived neurotrophic factor (BDNF) and nerve growth factor (NGF). These proteins have an important role in synaptic and neuronal growth, pruning, myelination, differentiation, and survival of neurons and are involved in the development of neurodegenerative diseases such as Alzheimer’s and dementia [11]. All of these changes lead to a progressive decline in cognitive faculty, memory difficulties and loss of executive function and speed of processing [3,8]. 

In response to this new social reality and the difficulties faced by this population group, it is necessary to search for new strategies to reduce the harmful effects of ageing on the brain and on vascular health. Thanks to the latest scientific findings, we know that the damage associated with ageing is multifactorial and that lifestyle habits play a fundamental role in promoting health and preventing the onset of multiple pathologies, especially regular physical exercise and the consumption of a balanced diet rich in bioactive compounds [12]. In this regard, dietary polyphenols have been shown in numerous studies to be effective in preventing cardiovascular disease [13] and cognitive decline [14]. Polyphenols are secondary metabolites in plants characterized by having more than one phenol group in their molecule structure. They exhibit a multitude of beneficial attributes for the organism, but the most important to take into consideration for the present work is their anti-ageing properties. Especially They have shown to be especially active in the brain, as they prevent the oxidation of neuronal fatty acids, reduce the damage caused by reactive oxygen and nitrogen species, and improve neurocognition by facilitating de novo protein synthesis in key sites and neurogenesis in dentate gyrus [14]. 

In particular, flavanols, present in foods such as fruit, cereals, cocoa and tea, have shown in several epidemiological studies to be very promising compounds in neurocognition. For example, the study by Mastroiacovo et al. (2015), in an ageing population without cognitive impairment, found that the supplementation with a cocoa drink containing 520 mg cocoa flavanols for two months significantly improved cognitive performance as measured by the Mini-Mental State Examination (MMSE), the Trail Making Test (TMT) A and B, and the Verbal Fluency Test (VFT), with a more significant effect in the case of the high flavonol drink [15]. While the study by Brickman et al. (2014), showed that older adults assigned to a three month high cocoa flavanol treatment (900-mg daily dose) had a significant increase in cerebral blood volume in the hippocampal region known as the dentate gyrus, also enhancing its function when measured by functional magnetic resonance imaging (fMRI) and by an image recognition test (ModBent task) [16]. The beneficial effect of flavonols are not only limited to cognitive health, as a large number of epidemiological studies have observed positive trends in the association between reduced cardiovascular risk and the consumption of different flavanol-rich foods such as tea [17], apples [18], red wine [19] and chocolate [20]. In fact, a meta-analysis conducted by Buitrago-Lopez et al. (2011) found that regular consumption of chocolate is associated with a 37% reduction in the risk of developing cardiovascular disease [21]. Similar results to those obtained by another meta-analysis conducted by Arab et al. (2009) on tea consumption found that those individuals who consume three cups a day had a 21% reduction in the risk of heart attacks compared to those who consumed less than one cup a day [22]. In fact, the European Food Safety Authority (EFSA) went so far as to state in a 2012 health claim that consumption of 200 mg of cocoa flavanols per day helps maintain endothelium-dependent vasodilation and normal blood flow [23]. This protective effect is often associated with the effects of flavanols on blood pressure, endothelial function and platelet reactivity, although the mechanisms involved have not been fully unraveled [24]. This is particularly important in view of the association between cardiovascular pathologies and cognitive deficit, with current evidence showing that 25–30% of heart attack survivors may develop immediate or delayed cognitive impairment or vascular dementia [25].

Other polyphenols that have also demonstrated beneficial effects on cognitive health are anthocyanins, natural pigments found in various fruits and vegetables, especially red fruits and red wines [26]. For instance, the study by Lamport et al. (2016) showed that the consumption of red grape juice high in anthocyanins (777 mg of total polyphenols) improved spatial memory and driving performance in both adults with intermediate cognitive impairment and healthy adult women [27]. Similarly, the study by Krikorian et al. (2010) in older adults with early cognitive deficits showed improved memory and learning measured by the Verbal Paired Associate Learning Test (V-PAL) and the California Verbal Learning Test (CVLT), and even a reduction in depressive symptoms measured by the Geriatric Depression Scale (GDS) following consumption of anthocyanin-rich blueberry juice (containing approximately 750 mg/L of chlorogenic acid and 900 mg) [28]. Similarly, Boespflug et al. (2018) found that in older adults with cognitive impairment, blueberry powder (with an anthocyanin content of 14.53 ± 0.04 mg) supplementation improved neuronal responses observed by MRI during memory challenges [29]. With regard to improving cardiovascular health, blueberry consumption has been shown to increase endothelium-dependent vasodilation in clinical trials in both healthy volunteers [30] and hypercholesteronemics [31]. Our group has also found that anthocyanins and their metabolites have an inhibitory effect on macrophage chemoadhesion protein 1 (MCP-1) secretion both in vitro [32] and in vivo [33], and also have an inhibitory effect on endothelial adhesion molecules (VCAM-1 and ICAM-1) in endothelial cells [32].

In view of the increase of cognitive deficits in the elderly and the need to mitigate their effects, the aim of this research was to study the activity of red fruit anthocyanins and cocoa flavanols on cognitive function, including neurocognitive tests and monitoring of sera neurotrophic factors like BDNF and NGF receptor. This with the aim of sustaining their inclusion in a balanced diet as a tool to reduce cognitive deficit in the elderly population.

## 2. Materials and Methods

### 2.1. Study Design and Ethical Considerations

This was a randomized, double-blinded, parallel-group study lasting 12 weeks. The volunteers were divided into three groups according to the type of product consumed: group 1 consumed a mixture of red berries (RB); group 2 consumed a cocoa powder rich in polyphenols (C) and group 3 consumed a mixture of cocoa and red berries (RB + C). For the design, preparation and supply of the products we have counted on the collaboration of the Salengei^®^ company (Barcelona, Spain). The randomization process was conducted based on age, sex, years of studies, BMI and smokers. To ensure the double-blinding of the study, each volunteer was assigned a numerical code and the dietary supplements were marked according to a code that was known only to the researcher in charge of data collection, with neither the volunteers nor the rest of those responsible for the study being aware of the group assignment.

In all, volunteers had to visit the ICTAN-CSIC Human Nutrition Unit three times for data and sample collection: a first visit for the interview and to confirm if volunteers met the inclusion criteria, the second visit was the start of the study (baseline values), and the third and last visit corresponded to the end of the intervention. At each visit, blood samples (approx. 20 mL), urine samples (first morning urine), anthropometric measurements (weight, height and waist circumference), blood pressure and three day diet records were taken. At each visit, the product to be consumed was provided and the rules of the study were outlined. In addition, volunteers were contacted by telephone every two weeks to check on compliance, resolve doubts and encourage completion of the study. The neurocognitive tests were carried out at the “Hospital 12 de Octubre” the same week as the sample collection. From the three day dietary recollections (which were to include two mid-weekdays and one weekend day), we also collected the frequency of consumption of the main sources of anthocyanins, flavanols, caffeine and theobromine in order to calculate their total daily intakes, knowing that the cocoa powder component of the study intervention included approximately 75 mg of theobromine and 3 mg of caffeine.

Body composition assessment of all patients was performed using standard anthropometric procedures. At each visit, weight was measured with a digital electronic scale (Seca Medical digital Scale 780, accuracy 0.100 g) and height with a stadiometer (Seca Medical digital Scale 220: 0.5 cm accuracy). From these anthropometric data, BMI, metabolic age and waist-to-height ratio (WHR) were calculated for each volunteer using the formulas most widely accepted by the scientific community for the study of this population group.

All personal data (biological samples and neuropsychological tests) were processed in accordance with Organic Law 15/1999, of 13 December, on the Protection of Personal Data, using codes to ensure confidentiality and guarantee anonymity from the moment of recruitment. The clinical trial was conducted in accordance with the principles of good clinical practice (Royal Decree 1090/2015 of 4 December) and the Declaration of Helsinki (https://www.wma.net/policies-post/wma-declaration-of-helsinki-ethical-principles-for-medical-research-involving-human-subjects/, accessed on 1 December 2016). The study was approved by the Ethics Committee for Clinical Research of the Hospital Universitario Puerta de Hierro-Majadahonda (Madrid, Spain) (Acta no 01.07), Hospital Universitario 12 de Octubre (Madrid, Spain) (Acta no17/117) and the Bioethics Committee of the Spanish National Research Council (CSIC). All subjects gave their written informed consent after receiving oral and written in-formation about the study. ClinicalTrials.gov trial registration NCT04348162.

### 2.2. Recruitment of Volunteers

Each study group consisted of 20 volunteers, making a total of 60 participants who successfully completed the trial. Volunteers were recruited through posters and oral communication in the Madrid area, as well as online and via dissemination through CSIC channels and social networks. We selected as potential candidates adults aged 50–75 years, both men and postmenopausal women (considered one year with amenorrhea). Participation in the study was voluntary.

Exclusion criteria were body mass index (BMI) <20 or >30, smokers of more than five cigarettes per day, menstruating women, familial hypercholesterolemia (serum triglycerides >250 mg/dL; or cardiovascular risk index total cholesterol/HDL-cholesterol >6), hypertension (systolic blood pressure >139 and diastolic >89 mmHg), diabetes, autoimmune diseases, chronic diseases (liver, kidney, etc.), treatment with cholesterol-lowering drugs, anticoagulants, regular intake of supplements/supplements with polyphenols, phytosterols, vitamins or minerals (volunteers were accepted if they were willing to stop treatment two months before the start of the study) and treatment with antibiotics in the last three months before the start of the study. Product aversion and a score below 28 on the screening cognitive test MMSE, and a score above 6 on the Functional Activities Questionnaire (FAQ) and over 10 on the Beck depression scale were also considered as exclusion criteria. 

### 2.3. Characterization of Cocoa and Red Berry Powders

All the products used in the present trial were kindly supplied by Salengei^®^ (Barcelona, Spain). The first one is a commercial semi-defatted cocoa powder from organic farming. It was labeled as sugar-, sweetener-, and emulsifier-free and as containing 7000 mg of flavanols, 2500 mg of theobromine, and 100 mg of caffeine in 100 g of cocoa powder, providing 200 mg of flavanols per day (flavanol richness ≥ 6%). The energy value of 100 g of the cocoa product was 353 kcal. The macronutrient composition per 100 g stated by the company is 11 g fat (of which 6.8 g saturated fat), 20 g carbohydrate (of which 2.45 g simple sugars), 32 g fiber and 27.8 g protein. In terms of micronutrients, the product was high in copper, iron, magnesium, potassium, phosphorus and zinc, with a particularly high content of copper, iron and magnesium. 

The red berry mixture was a combination of pure dried red (33.3%) and blackcurrants (33.3%), raspberries (16.7%) and blueberries (16.7%), which provided a daily intake of anthocyanins of 100 mg (flavanols richness = 1%). The energy value of 100 g of the red berry product was 355.5 kcal. The macronutrient composition per 100 g stated by the company was 2.7 g fat (of which 0.1 g saturated fat), 86.5 g carbohydrate (of which 35.4 g simple sugars), 39.9 g fiber and 7.3 g protein. In terms of micronutrients, the product had only 0.2 g of sodium. 

In both products, the method of use was to dissolve 1 tablespoon per day in water, vegetable milk, juice, yoghurt or cereals with breakfast. The size of each tablespoon of the product was explained to each volunteer before the start of the intervention.

### 2.4. Assessment of Cognitive Status

To check the suitability of the volunteers and to discard possible cognitive alterations, two neuropsychological tests were carried-out: the Folstein MMSE test for global cognitive evaluation [34] and the FAQ to assess the individual’s performance in daily activities [35]. The Beck Depression Scale [36] was also used to rule out possible depressive problems in our volunteers. 

During the follow-up of the study, a cognitive assessment was conducted in order to study memory performance and executive functions. For this purpose, the following neurocognitive tests were performed: TAVEC (Verbal Learning Test Spain-Complutense): A Spanish test similar to Rey’s “15 words test” which involves learning verbal elements. It consists of passing a list of 15 words that is applied five times to study the learning process, which is list A (Lissum). Subsequently, an interference list or B-list is applied, which we did not consider, in order for the volunteer to then remember list A to evaluate short-term memory (vlisARI), and after 20 min again to evaluate delayed or long-term memory (vlisARD). At the end, the volunteer would have to recognize from a list of words those that he/she did not remember (vRECTOT) [37]. Spatial recall test 10/36 BRB-N: This test consists of a matrix of 36 squares where 10 dots are drawn which the subject has to learn. They have three attempts to remember the correct location of the dots, in order to study visual learning (VmvisuTOT). Immediately after, the subject has to remember again the correct location of the dots for long-term recall (vmvisuRD) [38]. Wechsler Adult Intelligence Scale (WAIS) IV letters and numbers: In this task, a series of mixed numbers and letters are presented orally. The volunteer must repeat the numbers in ascending order and the letters in alphabetical order. Attention, concentration and working memory are tested at the end of the task (vLYNtot); the total score was used according to the test instructions [39]. STROOP Task: This test evaluates divided attention ability and resistance to interference. The test consists of three sheets, the first of which consists of reading the names of colours (green, red and blue) written in black ink (vStroopP), the second sheet consists of naming the print colour of some x’s (vStroopC), with the same colours as before; and the third sheet consists of the names of the colours written in a different colour (always the same colours as at the beginning). The subject has to name the colour by inhibiting the reading of the word (vStroopPC) [40]. Tower of London (TOL): This test consists of two boards with three sticks each, and each board has three balls (red, green and blue). One board is for the examiner, where he places the position of the balls that the patient has to reach using the other board. The volunteer starts always the problem with the balls in a given position and has to follow the instructions of the examiner’s board in as fewer movements as possible and as quickly as possible. There are two rules that must be followed: the first is that the sticks do not always hold all the balls and this must be respected, and the second rule is that the balls are picked up one at a time, always releasing one to pick up the other. The test consists of 12 planning problems. After the test, we evaluate the time it takes to start the first movement (vTOLstart), the total execution time (vTOLtot) and the total number of movements (vTOLmov) are measured [41]. WAIS-IV Symbol Digit Search: This test evaluates perceptual speed, accuracy and speed in processing simple visual information (vsym-bol) by recognizing symbols within a set [39]. WAIS-III number key: The task consists of completing, with the appropriate symbols, squares with a digit at the top. It assesses visual-motor speed and dexterity, pencil and paper handling and associative learning ability (vclaves) [42]. Number cancellation (ADAS-COG): The task consists of crossing out, within a time limit, certain numbers relative to other numbers. It measures vigilance, selective attention, visual processing speed and visual-motor coordination and speed (vcancel) [43]. WAIS-III Digits: This test consists in a series of numbers that the subject has to repeat, first in direct order (vdigDtot) and another time in indirect order (vdigItot). Each series of numbers is repeated twice, and the test ends when the subject fails to repeat either of the two series of the same length [42].

In order to better study the relationships between neurocognitive tests with biochemical parameters and polyphenol levels, the results of the different tests were combined according to three values of interest for our study; this transformation was done to simplify the calculations:

1. Sum of memory (SUMMEMO) result of adding together: (Lissum) + (vlisARI) + (vlisARD) + (vRECTOT) + (VmvisuTOT) + (vmvisuRD)
2. Sum of processing speed and attention (SUMVELOCITY) result of adding: (vStroopP) + (vStroopC) + (vStroopPC) + (vkeys) + (vcancel) + (vsymbol)
3. Working memory (SUMWOMEN) result of adding: (vLYNtot) + (vdigDtot) + (vdigItot)

### 2.5. Determination of Biochemical Parameters

For the determination of total polyphenols in urine, we used the protocol established by Hinojosa-Nogueira M. with modifications [44]. The standard curve was made from commercial gallic acid with serial dilutions from 300 to 4.7 µg/mL in ultrapure water. The urine samples were diluted by half. The diluted samples were centrifuged for 25 min at 4 °C and 13,200× *g*, pipetted onto a plate and 10 µL of Fast Blue rea-gent was added to set up the colour reaction and 10 µL of 5% NaOH to stop the reaction. After a two-hour incubation, the plate was read at an absorbance of 420 nm.

Urine creatinine was determined by a colorimetric reaction to normalise the data obtained for polyphenols [45]. The method was modified for determination in a 96-well plate. For this purpose, we started with the urine sample diluted 20-fold and pipetted 10 µL into each well of a 96-well flat-bottom plate. After that, we pipette 200 µL of 0.1% picric acid into each well and finally 15 µL of NaOH. After a 15 min incubation, the plate was read at an absorbance of 500 nm.

For the determination of BDNF and NGF-R, we used a sandwich ELISA (Enzyme-Linked Immuno-sorbent Assay) kit from Sigma-Aldrich, while for IL-6 we used the same type of ELISA kit from Invitrogen. In all three cases, the samples were brought to room temperature before insertion into the plate and were not diluted. In each well of the 96-well plate, 100 of the sample/standards were introduced. The assay was carried out according to the manufacturer’s specifications. After stopping the colour reaction with the stop solution, the plates were read at 450 nm. 

### 2.6. Statistical Analysis

Data analysis was carried out with IBM SPSS version 27 statistical software (SPSS, Inc., Chicago, IL, USA, 2020). The level of significance was set at *p* < 0.05. Values are expressed as mean and standard deviation (M ± SD). Data were examined for normal distribution with the Kolmogorov-Smirnov test. As a result of the normality test, a repeated measures ANOVA was applied when comparing the means of the three groups at the baseline visit with each other to observe the presence of significant differences between the different groups at baseline, and a repeated measures T-test to compare the results between baseline and after 12 weeks of intervention in each study group. In the case of the results obtained from the cognitive tests, where a negative result was obtained from the normality test, non-parametric tests were carried out for the analysis. Using a Wilcoxon rank test for related samples, in the case of comparing the results of the two visits and a Kruskal-Wallis test to compare the means of the three groups at the baseline. Bivariate correlations were also performed, segmented by sex, to test the association between BDNF, NGF-R and IL-6 with the different cognitive test results and creatinine-corrected polyphenol concentration. Scatter plots were also performed to graphically represent the relationship between both variables

## 3. Results

### 3.1. Baseline Population Characteristics

Table 1 summarizes the baseline parameters of our study population in total and in the three groups. As it can be seen, no significant differences were found in any of the groups with regards to age, sex, height, years of study and percentage of smokers. Only 20% of our study population were considered to be chronic smokers and the average number of years of study was approximately 13–14 years. 

### 3.2. Metabolic and Cardiovascular Parameters

As it can be seen in Table 2, we found no significant differences between groups or at the end of the intervention in any of the different markers of metabolic status. The average metabolic age was around 52 years, which is below the average age of our population (57 years).

With regard to blood pressure values (Table 3), no significant differences were observed between groups or after the intervention in diastolic and systolic blood pressure values.

However, significant differences were observed in heart rate values between the baseline and after 12 weeks in RB (*p* = 0.004), C (*p* = 0.007) and total (*p* = 0.001). All values of blood pressure are within the range of the values recognized as healthy by main medical institutions.

### 3.3. Anthropometric Parameters

The main anthropometric measurements carried out in the study are shown in Table 4. The average BMI of our population was 25 kg/m^2^ and the waist-to-height ratio (WHR) was 0.54, so we can consider that our population had low cardiovascular-risk values (between 0.53 and 0.57) [46]. Visceral fat was below 12 in all of the groups, thus no associated risk was shown. In relation to the significant differences between groups at the beginning of the study, we only observed significant differences in the values of fat (*p* = 0.022) between RB and C groups; and water percentage (*p* = 0.018) also in RB and C groups. We observed no significant differences in any of the markers (weight, fat, BMI, compartments, visceral fat and waist-to-height ratio) after the intervention. 

### 3.4. Polyphenol Levels Corrected by Creatinine

The statistical results of the creatinine-corrected polyphenol levels are shown in Table 5. There was no significant difference between the different groups. When looking at the differences within the groups, we could observe a significant increase in polyphenol intake in C-group (*p* = 0.03), while the RB-group remained close to significance (*p* = 0.059).

### 3.5. Biochemical Parameters

Table 6 represents the statistical results of different nerve growth factors (BDNF and NGF-R) and a parameter of inflammation (IL-6). As can be seen, the initial BDNF values present significant differences between RB and RB + C groups (*p* = 0.036), with no significant differences between groups in the rest of the parameters. Thus, we can confirm that, except for the values of BDNF, the rest of the groups started the study with similar levels. 

With respect to the significant differences within the groups, we only found significant differences in IL-6 between baseline and after 12 weeks of C (*p* ≤ 0.001), RB + C (*p* = 0.036) groups and in total (*p* = 0.001). In general, all of the products (except in the case of RB group) tend to increment the concentrations of IL-6, but these differences were more significant in C group.

### 3.6. Neurocognitive Tests Results

Table 7 represents the statistical results of the different neurocognitive test sums. We found no significant difference between the different study groups or within groups between baseline and after 12 weeks.

Upon individual examination of the different cognitive tests (Supplementary Table S1), we can observe a significant reduction after the intervention in both the time to start the TOL test (vTOLstart, *p* = 0.031) and the duration of the test in total (vTOLtot, *p* = 0.007), this significant reduction being more prominent in the RB + C group (*p* = 0.008). We also observed a reduction in the number of words required to recognise among other words in the TAVEC test (vRECTOT), with RB + C (*p* = 0.041) and an increase in perceptual speed, accuracy and speed in processing punctuation (vsym-bol) in total (*p* = 0.012).

### 3.7. Correlations between Biochemical Parameters, Polyphenol Intake and Cognitive Function

Table 8 shows the main results obtained after analyzing the correlations between the different sums of cognitive performance, polyphenol intake and biochemical parameters related to nerve growth and inflammation in men and women. 

**Figure 1:**
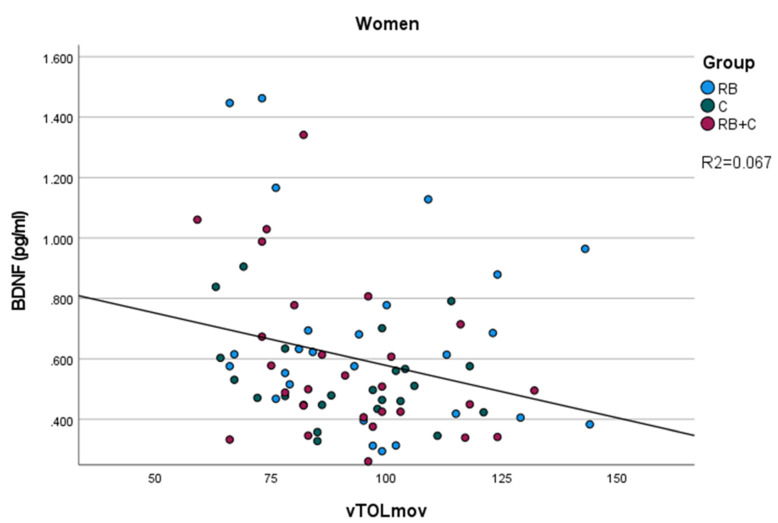
Correlations between levels of BDNF in blood and number of movements required to finish the tower of London (vTOLmov) in women.

In men, we found a positive correlation between polyphenol intake corrected for creatine and BDNF levels (*p* = 0.048), resulting in high polyphenol intake being associated with higher BDNF levels; and between number of movements required to finish the tower of London (vTOLmov) and NGF-R (*p* = 0.012). In women, these positive correlations are seen between SUMMEMO and SUMVELOCITY with NGF-R (*p* = 0.024 and *p* = 0.013 respectively). Consequently, we can deduce that high scores in these two tests are associated with a higher level of NGF. Additionally we found a negative correlation between the serum levels of BDNF and vTOLmov (*p* = 0.044), meaning that a higher levels of BDNF is associated with fewer number of movements required to finish the TOL test, as it can be seen in the Figure 1.

## 4. Discussion

Scientific evidence suggests that both flavanols [15,16] and anthocyanins [27,28,29] have neuroprotective qualities. Flavonoids, in general, seem to enhance neuronal function, protect vulnerable neurons and stimulate neuronal regeneration via interaction with neuronal intracellular signaling pathways involved in neuronal survival and differentiation, long-term potentiation (LTP), and memory. The mechanisms for the neuroprotective effect include increases in protective signaling, decreases in oxidative/inflammatory stress signaling and neurohormetic effects leading to the expression of genes that encode antioxidant enzymes, phase-2 enzymes, neurotrophic factors and cytoprotective proteins [47]. Focusing on their benefits on memory, the exact mechanisms by which flavonoids affect brain biochemistry are not precisely known, although it is speculated that they act as a promoter of new protein synthesis in neurons and thus induce morphological changes which have a direct influence on memory acquisition and consolidation. One of these changes is the activation of cAMP-response element-binding protein (CREB), a transcription factor which binds to the promoter regions of many genes associated with synapse re-modelling, synaptic plasticity and memory [47,48]. It also includes key components in the regulation of brain derivate neurotrophic factor (BDNF), a neurotrophin that is involved in the survival and function of neurons in the central nervous system and is an essential part of the formation of appropriate synaptic connections during development and for learning and memory in adults [49,50]. This upregulation mediated by CREB is well known in studies with animal models. For instance, in the study carried out by Williams et al. (2008) in aged rats it was observed that supplementation with a blueberry diet (including flavanols and anthocyanins) (2% *w*/*w*) for 12 weeks improved cognitive performance and memory in spatial work tasks, these changes being positively correlated with the activation of CREB and increases in both pro- and mature levels of BDNF in the hippocampus [50]. Another study, this time in young rats carried out by Rendeiro et al., also found that the supplementation for seven weeks with blueberry in the same concentration (2% *w*/*w*) also found an improvement in the spatial memory performance with an increase in total CREB activated by extracellular signal-related kinase (ERK1/2) and in pro- and mature levels of BDNF in the hippocampus. They also found an increase in BDNF mRNA in the dentate gyrus and CA1 areas of hipopocampi [51], areas in which BDNF is associated with adult neurogenesis and maturation, processes deeply associated with the establishment of new memories [52].

In our study, we did not find an improvement in serum BDNF levels with any of the interventions, with only a significant difference between RB and RB + C groups in the baseline. This differs from the results found in the literature, especially with the study carried out by Neshatdoust el al (2016) [53]. This work consists of a randomized, controlled, double-masked, crossover dietary intervention involving 40 cognitively normal elderly participants in which they consumed a high flavanol cocoa drink (494 mg flavanols) or a low flavanol cocoa drink (23 mg flavanols) for 28 days. After the intervention, a significantly better performance on the global executive function measures (*p* < 0.01), and an increase in BDNF levels (*p* < 0.01) was observed with the high flavanol cocoa drink in contrast with the low flavanol cocoa drink [53]. This could explain why in our trial we did not show any significant increase in BDNF serum levels and correlation with cognitive improvement, as our dietary dose was significantly lower than the high flavanol cocoa drink that showed a positive effect (200 mg vs 494 mg of flavanols per day). To our knowledge, this is the first human study on the effect of anthocyanins on BDNF concentrations and cognitive performance. However, it is important to mention the study carried out by Williams et al. (2008) as the most representative in this matter, displaying a positive effect in BDNF levels in rats after consumption of a supplement containing 267.2 μg/g of anthocyanins and 153.9 μg/g of flavanols, (~500 mg/day of lyophilized blueberry per day in supplemented group) [50].

On the other hand, we observed a negative correlation between BDNF levels, and the number of movements required to finish the Tower of London test(vTOLmov) was only significant in the case of women. This result confirms that higher levels of BDNF are associated with a better performance of the volunteers in this task, requiring less movements to finish the TOL. Moreover, we observed a better performance in this same test, with less time to start and finish the task after the intervention, more evident in the case of the combination of cocoa flavanols and red berry anthocyanins. Our study confirms that flavonoid ingestion improves the executive function, which is in line with multiple studies describing the positive effects of flavanols and anthocyanins in preventing mind impairment [15,16,27,28,29,54,55]. For example, Miller et al. (2018) conducted a randomized, double-blind, placebo-controlled trial with 37 healthy volunteers aged 60–75 in which, after a 90 day intervention with 24 g per day of freeze-dried blueberries (containing 19.2 mg/g anthocyanins), the intervention group showed reduced switch stimuli errors (measure of executive function) on task switching test compared to the controls [56]. In the meta-analysis carried out by Gardener et al. (2021) they examined 28 papers published between 2014 and 2020. The authors concluded that in most of the studies they observed that both flavanols and anthocyanins positively modulate various cognitive domains, including executive function [54]. 

Additionally, we observed a positive correlation between polyphenol levels corrected by creatinine and BDNF in male subjects, showing that higher levels of polyphenols corrected by creatinine matched with higher levels of BDNF levels in serum. As far as we know, there is no previous study showing a correlation between the levels of polyphenols and blood BDNF levels. However, this is in accordance with our first hypothesis being that flavonoids, in this case flavanols and anthocyanins, have a positive effect in neurocognition by upregulating BDNF and maintaining cognitive health [54,55]. The absence of this correlation in women may be explained by the estrogenic-gut microbiota interaction, as was shown by Xu et al. (1995) [57]. However, more recently, Mayneris-Perxachs et al. (2020) showed that the microbiota of postmenopausal women is more similar to that of men than to that of premenopausal women, and that obesity eliminates the differences between men and women, both pre- and postmenopausal [58]. This issue should be the focus of future work in this same context. In regard to polyphenol levels corrected by creatinine, we only found a significant increase in the group corresponding to the cocoa supplementation. This could be explained because among all dietary flavonoids, flavanols tend to be more bioavailable than anthocyanins [59], and it might be possible that there exists an interaction in terms of bioavailability between flavanols and anthocyanins that interfered in the absorption of flavanols when ingested as a combination. 

In our study population, no statistically significant differences were shown between groups as for gender, age, years of study, height and percentage of smokers. This confirms that our parallel groups were well balanced and homogeneous, and that the randomization was carried-out correctly. Moreover, we did not show any statistically significant change in any anthropometric, metabolic or vascular parameter through the study, showing that none of our positive results were associated with an improvement in any of the anthropometric or metabolic parameters.

Additionally to BDNF, we studied the levels of NGF-R and IL-6 in blood. Nerve growth factor (NGF) is one of the best known neurotrophins which acts on central cholinergic neurons as well as subsets of peripheral neurons regulating growth, maintenance, proliferation, and survival. The biological functions of NGF are mediated through two classes of cell surface receptors, the Trk receptors and the p75 neurotrophin receptor (p75NTR) [60,61]. Several studies have shown an increase in NGF receptor concentrations with various neurodegenerative diseases. For instance, in the study conducted by Messripour et al. a significant increase of TNGFR levels in urine samples was observed in dementia patients compared to healthy subjects [62]. In our study, we did not find any statistically significant differences between the levels of NGF-R in blood between groups and between baseline and after 12 weeks intervention. However, we observed a positive correlation in women between NGF-R levels and the two cognitive test sums SUMMEMO and SUMVELOCITY and in men with vTOLmov. The parameter known as SUMVELOCITY is a sum of different neurocognitive tests focusing on the time taken by the subject to complete the tests. From the statistical correlations test, we can conclude that higher levels of NGF-R are correlated with worst performances in SUMVELOCITY and vTOLmov. We also found a positive correlation in this same population between the punctuations in memory task (SUMMEMO) and the concentrations of NGF-R. This seems to be contradictory and we cannot find a possible explanation in the literature, so future research is needed to determine how circulating levels of NGF-R are affected by flavonoids and how this relates to cognitive impairment in an ageing population.

As for IL-6, we found an increase after the 12-week intervention that was statistically significant for the cocoa flavanols (C) group and the combined red-berry anthocyanins and cocoa flavanols (RB + C) group. IL-6 can be secreted by both immune and non-immune cells including neurons, but its receptor is only found on restricted subsets of cell types such as hepatocytes or the microglia [63]. IL-6 acts both as an anti-inflammatory in muscle tissue (myokine) and as a pro-inflammatory cytokine [64]. In the brain, levels of Il-6 tend to be low [63]. However, as has been shown in multiple studies, including a meta-analysis by Swardfager et al. (2020), levels of IL-6 seem to be elevated in neurodegenerative diseases such as Alzheimer’s and Parkinson’s diseases, revealing an inflammatory response in these conditions [65]. In fact, increased levels of IL-6 with low BDNF levels in the blood have been observed in studies with mayor depression [66,67], and a negative correlation between BDNF levels and IL-6 expression was found in patients with first-episode psychosis [68], suggesting an inflammation mediated decrease in BDNF expression. Moreover, it is worth mentioning that IL-6 levels tend to increase with age [69]. In our study, we did find a nearly significant correlation between the age of the volunteers and IL-6 levels (*p* = 0.082). Additionally, other studies have shown that a dietary intervention with flavonoids tends to maintain IL-6 levels unaffected. This was the case of the trial carried out by Kent et al. (2017) in patients with mild to moderate Alzheimer’s disease who were treated with cherry juice high in anthocyanins for 12 weeks [70]. Moreover, some authors have shown an increase in IL-6 levels with flavonoid-rich fruits and vegetables [71].

Therefore, our study showed an improvement in executive function with the dietary intervention that failed to show an association with increased BDNF levels. One should take into consideration that flavanols and anthocyanins not only directly affect neurons, as multiple studies have shown in the past, but that the consumption of foods rich in flavonoids improves the glucoregulatory control via improved insulin sensitivity [53] and cerebral blood flow by increasing the bioavailability of nitric oxide [16,24,30,31,54], resulting in improved cognitive performance. Future work should focus on the metabolic and cerebrovascular markers that might explain our present results with cocoa flavanols and red-berry anthocyanins. However, it should be noted that our study had two important limitations in that the number of subjects was slightly low considering that it was a parallel study and that there was no control group included.

## 5. Conclusions

A combination of both cocoa flavanols and red berry anthocyanins improve executive function of healthy aged subjects, enhancing performance on the neurocognitive test TOL. 

This improvement is not associated with changes in the levels of BDNF or NGF-R. However, we found a correlation between high BDNF levels and a better task performance (fewer movements required to finish the TOL) in women. Additionally, we found positive correlations between higher levels of NGF-R and the time requirement to finish a working memory test in both women and men.

Further studies are needed to better understand the mechanisms implied in the potential neurocognitive protective effect of cocoa flavanols and red-berry anthocyanins.

## Figures and Tables

**Table 1:** Demographics and characteristics of the participants at enrollment in each group.

	Total	RB	C	RB + C	*p*-Value
N	59	20	20	19	
Females	71.2%	60%	85%	68.4%	0.215
Age (years)	57.8 ± 6.94	56.4 ± 4.14	59.15 ± 9.08	57.84 ± 6.76	0.463
Years of study	13.57 ± 4.32	13.37 ± 3.63	14.95 ± 3.70	12.32 ± 5.25	0.224
Height (m)	1.65 ± 0.08	1.67 ± 0.1	1.62 ± 0.08	1.66 ± 0.06	0.141
Smokers (%)	20%	15%	20%	26.3%	0.795

Data expressed as mean ± standard deviation (SD); significant difference at *p*-value < 0.05.

**Table 2:** Metabolic parameters in each group and by visit.

	RB			C			RB + C			Total			*p*-Value **
	Baseline	12 w	*p*-Value *	Baseline	12 w	*p*-Value *	Baseline	12 w	*p*-Value *	Baseline	12 w	*p*-Value *	
Basal metabolism (Kcal)	2354 ± 526.69	2325.32 ± 491.9	0.81	2110.05 ± 398.15	2134.85 ± 420.26	0.182	2284.89 ± 323.67	2276.58 ± 315.9	0.554	2249.05 ± 431.5	2243.67 ± 416.59	0.492	0.185
Metabolic age	47.15 ± 9.89	47.74 ± 10.96	0.839	55.75 ± 14.95	56.55 ± 15.27	0.57	51.37 ± 11.15	51.42 ± 11.08	0.955	51.42 ± 12.51	51.98 ± 12.96	0.577	0.093

* Significance within groups, ** significance between groups; data expressed as mean ± standard deviation (SD); significant difference at *p*-value < 0.05.

**Table 3:** Blood pressure values in each group and by visit.

	RB			C			RB + C			Total			*p*-Value **
	Baseline	12 w	*p*-Value *	Baseline	12 w	*p*-Value *	Baseline	12 w	*p*-Value *	Baseline	12 w	*p*-Value *	
Systolic pressure (mmHg)	123.1 ± 16.3	124.9 ± 16.3	0.336	126.2 ± 20.8	122.2 ± 18.5	0.216	118.4 ± 16.3	118.8 ± 16.1	0.888	122.7 ± 17.9	122 ± 16.9	0.665	0.399
Diastolic preassure (mmHg)	83 ± 11.1	83.4 ± 13.5	0.709	83.6 ± 12.8	81.2 ± 8.4	0.181	78.4 ± 10.8	80 ± 10.4	0.426	81.7 ± 11.7	81.5 ± 10.8	0.871	0.324
Heart rate (lpm)	64.3 ± 9.79	69.97 ± 12.28	0.004	67.77 ± 10.71	73.06 ± 12.21	0.007	65.08 ± 7.88	66.99 ± 9.51	0.342	65.73 ± 9.51	70.06 ± 11.5	<0.001	0.49

* Significance within groups, ** significance between groups; data expressed as mean ± standard deviation (SD); significant difference at *p*-value < 0.05.

**Table 4:** Anthropometric parameters in each group and by visit.

	RB			C			RB + C			Total			*p*-Value **
	Baseline	12 w	*p*-Value *	Baseline	12 w	*p*-Value *	Baseline	12 w	*p*-Value *	Baseline	12 w	*p*-Value *	
Weight (kg)	71 ± 13.48	69.07 ± 12.8	0.767	69.07 ± 13.04	69.31 ± 13.41	0.428	70.86 ± 9.47	71.25 ± 9.54	0.327	70.3 ± 12	69.88 ± 11.87	0.201	0.856
BMI (kg/m^2^)	25.24 ± 2.62	25.03 ± 2.45	0.829	26.23 ± 4.44	26.38 ± 4.52	0.13	25.91 ± 3.38	25.89 ± 3.04	0.909	25.79 ± 3.52	25.78 ± 3.46	0.412	0.668
Body Fat (%)	30.72 ± 7.86 ^a^	30.59 ± 7.37	0.889	34.37 ± 7.7 ^b^	33.92 ± 7.47	0.539	30.03 ± 6.96 ^ab^	30.23 ± 7.06	0.507	30.72 ± 7.86	30.59 ± 7.37	0.754	0.022
Muscle (%)	48.46 ± 12.19	48.31 ± 10.87	0.459	42.73 ± 8.73	43.33 ± 9.21	0.237	47.24 ± 7.27	47.06 ± 7.21	0.565	46.12 ± 9.81	46.18 ± 9.31	0.294	0.152
Bone (%)	2.6 ± 0.58	2.57 ± 0.54	0.881	2.29 ± 0.44	2.32 ± 0.46	0.267	2.52 ± 0.37	2.5 ± 0.36	0.42	2.47 ± 0.48	2.46 ± 0.46	0.663	0.109
Water (%)	52.25 ± 5.23 ^a^	52.35 ± 4.42	0.895	47.53 ± 5.44 ^b^	47.74 ± 5.13	0.664	50.71 ± 4.84 ^ab^	50.54 ± 4.85	0.462	50.15 ± 5.47	50.17 ± 5.11	0.872	0.018
Visceral fat	8.11 ± 3.05	8 ± 2.53	0.899	8.7 ± 3.56	9.4 ± 3.56	0.1	9.11 ± 3.6	8.95 ± 3.31	0.187	8.59 ± 3.24	8.83 ± 3.3	0.304	0.566
Waist (cm)	86.78 ± 10.44	87.74 ± 9.58	0.187	89.85 ± 12.42	89.2 ± 12.13	0.49	89.47 ± 12.16	89.68 ± 10.64	0.812	88.69 ± 11.58	88.88 ± 10.7	0.581	0.666
WHR	0.52 ± 0.05	0.53 ± 0.04	0.233	0.55 ± 0.08	0.55 ± 0.08	0.438	0.54 ± 0.07	0.51 ±	0.387	0.54 ± 0.07	0.53 ± 0.09	0.442	0.257

* Significance within groups, ** significance between groups; data expressed as mean ± standard deviation (SD); significant difference at *p*-value < 0.05; different letters indicate statistically significant differences between groups; BMI: Body mass index; WHR: Waist to height ratio.

**Table 5:** Creatinine-corrected polyphenol levels in each group and by visit.

	RB			C			RB + C			Total			*p*-Value **
	Baseline	12 w	*p*-Value *	Baseline	12 w	*p*-Value *	Baseline	12 w	*p*-Value *	Baseline	12 w	*p*-Value *	
polyphenol/ creatinine (mg/mL)	120.16 ± 55.53	151.9 ± 62.3	0.059	158.78 ± 60.49	199.7 ± 86.65	0.03	119.57 ± 40.65	130.96 ± 64.74	0.082	156.72 ± 83.58	180.06 ± 84.62	0.462	0.092

* Significance within groups, ** significance between groups; data expressed as mean ± standard deviation (SD); significant difference at *p*-value < 0.05.

**Table 6:** Biochemical parameters of nerve growth and inflammation in each group and by visit.

	RB			C			RB + C			Total			*p*-Value **
	Baseline	12 w	*p*-Value *	Baseline	12 w	*p*-Value *	Baseline	12 w	*p*-Value *	Baseline	12 w	*p*-Value *	
BDNF (pg/mL)	0.77 ± 0.37 ^a^	0.66 ± 0.34	0.384	0.58 ± 0.16 ^ab^	0.56 ± 0.19	0.33	0.54 ± 0.24 ^b^	0.64 ± 0.31	0.301	0.63 ± 0.28	0.62 ± 0.28	0.888	0.036
NGF-R (pg/mL)	80.23 ± 63.37	97.59 ± 104.19	0.618	63.81 ± 35.49	77.5 ± 61.44	0.46	80.91 ± 66.84	83.03 ± 64.27	0.872	74.98 ± 56.25	86.04 ± 77.74	0.399	0.651
IL-6 (pg/mL)	0.7 ± 0.42	0.95 ± 0.35	0.195	0.35 ± 0.28	0.85 ± 0.35	<0.001	0.52 ± 0.4	1.41 ± 1.11	0.036	0.51 ± 0.37	1.08 ± 0.71	0.001	0.374

* Significance within groups, ** significance between groups; data expressed as mean ± standard deviation (SD); significant difference at *p*-value < 0.05; different letters indicate statistically significant differences between groups; BDNF: Brain-derived neurotrophic factor; NGF-R: Nerve growth factor receptor.

**Table 7:** Statistical results of the different summatives for each test in each group and by visit.

	RB			C			RB + C			Total			*p*-Value **
	Baseline	12 w	*p*-Value *	Baseline	12 w	*p*-Value *	Baseline	12 w	*p*-Value *	Baseline	12 w	*p*-Value *	
SUMMEMO	148.26 ± 12.17	150.74 ± 17.18	0.587	146.7 ± 16.26	150 ± 19	0.401	149.7 ± 17.82	144.63 ± 20.12	0.513	148.22 ± 15.43	148.48 ± 18.68	0.636	0.911
SUMWOMEN	34.58 ± 5.36	33.32 ± 7.36	0.434	33.85 ± 6.05	35.2 ± 7.22	0.211	33.6 ± 6.75	33.21 ± 4.78	0.831	34 ± 6	33.93 ± 6.53	0.708	0.535
SUMVELOCITY	310.89 ± 55.42	319.68 ± 58.31	0.888	306.75 ± 53.34	310.1 ± 51.61	0.601	287.45 ± 53.23	302.37 ± 67.01	0.231	301.54 ± 54.03	310.71 ± 58.556	0.294	0.232

* Significance within groups, ** significance between groups; data expressed as mean ± standard deviation (SD); significant difference at *p*-value < 0.05; SUMMEMO: Sum of memory; SUMWOMEN: Working memory; SUMVELOCITY: Sum of processing speed and attention.

**Table 8:** Sex-segmented bivariate correlations between biochemical parameters and the sum of cognitive tests and polyphenol intakes.

	Men						Women					
	BDNF		NGF-R		IL-6		BDNF		NGF-R		IL-6	
	R	*p*-Value	R	*p*-Value	R	*p*-Value	R	*p*-Value	R	*p*-Value	R	*p*-Value
SUMMEMO	0.058	0.768	−0.032	0.884	−0.041	0.889	0.021	0.855	0.279	0.024	−0.072	0.71
SUMWOMEN	0.047	0.812	−0.237	0.276	−0.15	0.608	0.119	0.304	0.183	0.144	−0.24	0.21
SUMVELOCITY	0.086	0.662	−0.227	0.298	−0.127	0.666	0.053	0.648	0.307	0.013	−0.304	0.109
vTOLmov	−0.144	0.466	0.516	0.012	−0.204	0.485	−0.229	0.044	−0.007	0.953	−0.061	0.755
Polyphenol	0.391	0.048	0.156	0.487	−0.419	0.154	−0.038	0.749	0.091	0.497	0.21	0.313

Data expressed as mean ± standard deviation (SD); significant difference at *p*-value < 0.05; SUMMEMO: Sum of memory; SUMWOMEN: Working memory; SUMVELOCITY: Sum of processing speed and attention; vTOLmov: number of movements to finish the tower of London.

## Data Availability

Data is contained within the article and Supplementary Materials.

## Supplementary Materials

The following are available online at https://www.mdpi.com/article/10.3390/nu14010001/s1, Supplementary Table S1: Statistical results of the different neurocognitive test for each group and by visit.

## References

[1] (2019). United Nations World Population Ageing. https://www.un.org/en/development/desa/population/publications/pdf/ageing/WorldPopulationAgeing2019-Highlights.

[2] (2019). Ageing Europe—Looking at the Lives of Older People in The EU—Eurostat 2019 Report. https://www.age-platform.eu/publications/ageing-europe-looking-lives-older-people-eu-eurostat-2019-report.

[3] Han L.K.M., Verhoeven J.E., Tyrka A.R., Penninx B.W., Wolkowitz O.M., Månsson K.N., Lindqvist D., Boks M.P., Révész D., Mellon S.H. (2019). Accelerating research on biological aging and mental health: Current challenges and future directions. Psychoneuroendocrinology.

[4] Fernandes M., Wan C., Tacutu R., Barardo D., Rajput A., Wang J., Thoppil H., Thornton D., Yang C., Freitas A. (2016). Systematic analysis of the gerontome reveals links between aging and age-related diseases. Hum. Mol. Genet..

[5] Petersen R.C., Lopez O., Armstrong M.J., Getchius T.S.D., Ganguli M., Gloss D., Gronseth G.S., Marson D., Pringsheim T., Day G.S. (2018). Practice guideline update summary: Mild cognitive impairment: Report of the guideline de-velopment, dissemination, and implementation subcommittee of the american academy of neurology. Neurology.

[6] Harada C.N., Natelson Love M.C., Triebel K.L. (2013). Normal cognitive aging. Clin. Geriatr. Med..

[7] Lockhart S., DeCarli C. (2014). Structural imaging measures of brain aging. Neuropsychol. Rev..

[8] Juan S.M.A., Adlard P.A. (2019). Ageing and cognition. Subcell Biochem..

[9] Fjell A.M., Walhovd K.B. (2010). Structural brain changes in aging: Courses, causes and cognitive consequences. Rev. Neurosci..

[10] Różycka A., Liguz-Lecznar M. (2017). The space where aging acts: Focus on the GABAergic synapse. Aging Cell.

[11] Budni J., Bellettini-Santos T., Mina F., Garcez M.L., Zugno A.I. (2015). The involvement of BDNF, NGF and GDNF in aging and Alz-heimer’s disease. Aging Dis..

[12] Marsman D., Belsky D.W., Gregori D., Johnson M.A., Dog T.L., Meydani S., Pigat S., Sadana R., Shao A., Griffiths J.C. (2018). Healthy ageing: The natural consequences of good nutrition—A conference report. Eur. J. Nutr..

[13] Serino A., Salazar G. (2018). Protective role of polyphenols against vascular inflammation, aging and cardiovascular disease. Nutrients.

[14] Devi S.A., Chamoli A. (2020). Polyphenols as an effective therapeutic intervention against cognitive decline during normal and pathological brain aging. Adv. Exp. Med. Biol..

[15] Mastroiacovo D., Kwik-Uribe C., Grassi D., Necozione S., Raffaele A., Pistacchio L., Righetti R., Bocale R., Lechiara M.C., Marini C. (2015). Cocoa flavanol consumption improves cognitive function, blood pressure control, and metabolic profile in elderly subjects: The cocoa, cognition, and aging (cocoa) study—A randomized controlled trial. Am. J. Clin. Nutr..

[16] Brickman A.M., Khan U.A., Provenzano F.A., Yeung L.K., Suzuki W., Schroeter H., Wall M., Sloan R.P., Small S.A. (2014). Enhancing dentate gyrus function with dietary flavanols improves cognition in older adults. Nat. Neurosci..

[17] Ivey K.L., Lewis J.R., Prince R.L., Hodgson J.M. (2013). Tea and non-tea flavonol intakes in relation to atherosclerotic vascular disease mortality in older women. Br. J. Nutr..

[18] Knekt P., Jarvinen R., Reunanen A., Maatela J. (1996). Flavonoid intake and coronary mortality in Finland: A cohort study. BMJ.

[19] Mink P.J., Scrafford C.G., Barraj L.M., Harnack L., Hong C.-P., Nettleton J.A., Jacobs D.R. (2007). Flavonoid intake and cardiovascular disease mortality: A prospective study in postmenopausal women. Am. J. Clin. Nutr..

[20] Lewis J.R., Prince R.L., Zhu K., Devine A., Thompson P.L., Hodgson J.M. (2010). Habitual chocolate intake and vascular disease: A pro-spective study of clinical outcomes in older women. Arch. Intern. Med..

[21] Lopez A.B., Sanderson J., Johnson L., Warnakula S., Wood A., Di Angelantonio E., Franco O. (2011). Chocolate consumption and cardiometabolic disorders: Systematic review and meta-analysis. BMJ.

[22] Arab L., Liu W., Elashoff D. (2009). Green and black tea consumption and risk of stroke: A meta-analysis. Stroke.

[23] EFSA Panel on Dietetic Products, Nutrition and Allergies (NDA) (2012). Scientific opinion on the substantiation of a health claim related to cocoa flavanols and maintenance of normal endothelium-dependent vasodilation pursuant to Article 13 (5) of Regulation (EC) No 1924/2006. EFSA J..

[24] Bondonno C.P., Croft K.D., Ward N., Considine M.J., Hodgson J.M. (2015). Dietary flavonoids and nitrate: Effects on nitric oxide and vascular function. Nutr. Rev..

[25] Kalaria R.N., Akinyemi R., Ihara M. (2016). Stroke injury, cognitive impairment and vascular dementia. Biochim. Biophys. Acta (BBA)-Mol. Basis Dis..

[26] Mattioli R., Francioso A., Mosca L., Silva P. (2020). Anthocyanins: A comprehensive review of their chemical properties and health effects on cardiovascular and neurodegenerative diseases. Molecules.

[27] Lamport D.J., Lawton C.L., Merat N., Jamson H., Myrissa K., Hofman D., Chadwick H.K., Quadt F., Wightman J.D., Dye L. (2016). Concord grape juice, cognitive function, and driving performance: A 12-wk, placebo-controlled, randomized crossover trial in mothers of preteen children. Am. J. Clin. Nutr..

[28] Krikorian R., Shidler M.D., Nash T.A., Kalt W., Vinqvist-Tymchuk M.R., Shukitt-Hale B., Joseph J.A. (2010). Blueberry supplementation improves memory in older adults. J. Agric. Food Chem..

[29] Boespflug E.L., Eliassen J.C., Dudley J.A., Shidler M.D., Kalt W., Summer S.S., Stein A.L., Stover A.N., Krikorian R. (2018). Enhanced neural activation with blueberry supplementation in mild cognitive impairment. Nutr. Neurosci..

[30] Rodriguez-Mateos A., Rendeiro C., Bergillos-Meca T., Tabatabaee S., George T.W., Heiss C., Spencer J.P. (2013). Intake and time de-pendence of blueberry flavonoid-induced improvements in vascular function: A randomized, controlled, double-blind, crossover intervention study with mechanistic insights into biological activity. Am. J. Clin. Nutr..

[31] Zhu Y., Xia M., Yang Y., Liu F., Li Z., Hao Y., Mi M., Jin T., Ling W. (2011). Purified anthocyanin supplementation improves endothelial function via NO-cGMP activation in hypercholesterolemic individuals. Clin. Chem..

[32] García-Alonso M., Rimbach G., Rivas-Gonzalo J.C., de Pascual-Teresa S. (2004). Antioxidant and cellular activities of anthocyanins and their corresponding vitisins A—Studies in platelets, monocytes, and human endothelial cells. J. Agric. Food Chem..

[33] Garcia-Alonso M., Minihane A.-M., Rimbach G., Rivas-Gonzalo J.-C., De Pascual-Teresa S. (2009). Red wine anthocyanins are rapidly absorbed in humans and affect monocyte chemoattractant protein 1 levels and antioxidant capacity of plasma. J. Nutr. Biochem..

[34] Blesa R., Pujol M., Aguilar M., Santacruz P., Bertran-Serra I., Hernández G., Sol J.M., Peña-Casanova J., NORMACODEM Group (2001). NORMAlisation of cognitive and functional instruments for DEMentia. Clinical validity of the ‘mini-mental state’ for Spanish speaking communities. Neuropsychologia.

[35] Olazarán J., Mouronte P., Bermejo F. (2005). Clinical validity of two scales of instrumental activities in Alzheimer’s disease. Neurologia.

[36] Beck A.T., Steer R.A., Ball R., Ranieri W.F. (1996). Comparison of beck depression inventories-IA and-II in psychiatric outpatients. J. Personal. Assess..

[37] Benedet M.J. (2014). Versión paralela del test de aprendizaje verbal España-Complutense (TAVEC). Rev. Neurol..

[38] Boringa J.B., Lazeron R.H., Reuling I.E., Adèr H.J., Pfennings L., Lindeboom J., de Sonneville L.M., Kalkers N.F., Polman C.H. (2001). The brief repeatable battery of neuropsychological tests: Normative values allow application in multiple sclerosis clinical practice. Mult. Scler..

[39] Holdnack J.A., Zhou X., Larrabee G.J., Millis S.R., Salthouse T.A. (2011). Confirmatory factor analysis of the WAIS-IV/WMS-IV. Assessment.

[40] Scarpina F., Tagini S. (2017). The Stroop color and word test. Front. Psychol..

[41] Shallice T. (1982). Specific impairments of planning. Philos. Trans. R. Soc. Lond. B Biol. Sci..

[42] Wechsler D. (1997). wais-iii Administration and Scoring Manual.

[43] Schultz R., Siviero M., Bertolucci P. (2001). The cognitive subscale of the “Alzheimer’s Disease Assessment Scale” in a Brazilian sample. Braz. J. Med. Biol. Res..

[44] Hinojosa-Nogueira D., Muros J., Rufián-Henares J.A., Pastoriza S. (2017). New method to estimate total polyphenol excretion: Comparison of Fast Blue BB versus Folin–Ciocalteu performance in urine. J. Agric. Food Chem..

[45] Roura E., Andrés-Lacueva C., Estruch R., Lamuela-Raventós R.M. (2006). Total polyphenol intake estimated by a modified Folin-Ciocalteu assay of urine. Clin. Chem..

[46] Schneider H.J., Friedrich N., Klotsche J., Pieper L., Nauck M., John U., Dörr M., Felix S., Lehnert H., Pittrow D. (2010). The predictive value of different measures of obesity for incident cardiovascular events and mortality. J. Clin. Endocrinol. Metab..

[47] Spencer J.P.E. (2009). Flavonoids and brain health: Multiple effects underpinned by common mechanisms. Genes Nutr..

[48] Spencer J.P.E. (2009). The impact of flavonoids on memory: Physiological and molecular considerations. Chem. Soc. Rev..

[49] Thomas K., Davies A. (2005). Neurotrophins: A Ticket to Ride for BDNF. Curr. Biol..

[50] Williams C., El Mohsen M.A., Vauzour D., Rendeiro C., Butler L.T., Ellis J.A., Whiteman M., Spencer J.P. (2008). Blueberry-induced changes in spatial working memory correlate with changes in hippocampal CREB phosphorylation and brain-derived neurotrophic factor (BDNF) levels. Free. Radic. Biol. Med..

[51] Rendeiro C., Vauzour D., Kean R.J., Butler L.T., Rattray M., Spencer J.P.E., Williams C.M. (2012). Blueberry supplementation induces spatial memory improvements and region-specific regulation of hippocampal BDNF mRNA expression in young rats. Psychopharmacology.

[52] Zhao C., Deng W., Gage F.H. (2008). Mechanisms and functional implications of adult neurogenesis. Cell.

[53] Neshatdoust S., Saunders C., Castle S.M., Vauzour D., Williams C., Butler L., Lovegrove J.A., Spencer J.P.E. (2016). High-flavonoid intake induces cognitive improvements linked to changes in serum brain-derived neurotrophic factor: Two randomised, controlled trials. Nutr. Health Aging.

[54] Gardener S.L., Rainey-Smith S.R., Weinborn M., Bondonno C.P., Martins R.N. (2021). Intake of products containing anthocyanins, fla-vanols, and flavanones, and cognitive function: A narrative review. Front. Aging Neurosci..

[55] Zhang J., Wu J., Liu F., Tong L., Chen Z., Chen J., He H., Xu R., Ma Y., Huang C. (2019). Neuroprotective effects of anthocyanins and its major component cyanidin-3-O-glucoside (C3G) in the central nervous system: An outlined review. Eur. J. Pharmacol..

[56] Miller M.G., Hamilton D.A., Joseph J.A., Shukitt-Hale B. (2018). Dietary blueberry improves cognition among older adults in a ran-domized, double-blind, placebo-controlled trial. Eur. J. Nutr..

[57] Xu X., Harris K.S., Wang H.-J., Murphy P.A., Hendrich S. (1995). Bioavailability of soybean isoflavones depends upon gut microflora in women. J. Nutr..

[58] Mayneris-Perxachs J., Arnoriaga-Rodríguez M., Luque-Córdoba D., Priego-Capote F., Pérez-Brocal V., Moya A., Burokas A., Maldonado R., Fernández-Real J.M. (2020). Gut microbiota steroid sexual dimorphism and its impact on gonadal steroids: Influences of obesity and menopausal status. Microbiome.

[59] Viskupicova J., Ondrejovic M., Sturdik E. (2008). Bioavailability and metabolism of flavonoids. J. Food Nutr. Res..

[60] Wiesmann C., De Vos A. (2001). Nerve growth factor: Structure and function. Cell. Mol. Life Sci..

[61] Lang U.E., Gallinat J., Danker-Hopfe H., Bajbouj M., Hellweg R. (2003). Nerve growth factor serum concentrations in healthy human volunteers: Physiological variance and stability. Neurosci. Lett..

[62] Messripour M., Nazarian A., Mesripour A., Mohammadi I. (2015). Nerve growth factor receptors in dementia. Turk. J. Med. Sci..

[63] Rothaug M., Becker-Pauly C., Rose-John S. (2016). The role of interleukin-6 signaling in nervous tissue. Biochim. Biophys. Acta (BBA)-Mol. Cell Res..

[64] Mihara M., Hashizume M., Yoshida H., Suzuki M., Shiina M. (2012). IL-6/IL-6 receptor system and its role in physiological and pathological conditions. Clin. Sci..

[65] Swardfager W., Lanctôt K., Rothenburg L., Wong A., Cappell J., Herrmann N. (2010). A Meta-Analysis of Cytokines in Alzheimer’s Disease. Biol. Psychiatry.

[66] Ting E.Y.-C., Yang A.C., Tsai S.-J. (2020). Role of interleukin-6 in depressive disorder. Int. J. Mol. Sci..

[67] Jehn C., Becker B., Flath B., Nogai H., Vuong L., Schmid P., Lüftner D. (2015). Neurocognitive function, brain-derived neurotrophic factor (BDNF) and IL-6 levels in cancer patients with depression. J. Neuroimmunol..

[68] Mondelli V., Cattaneo A., Murri M.B., Di Forti M., Handley R., Hepgul N., Miorelli A., Navari S., Papadopoulos A.S., Aitchison K.J. (2011). Stress and inflammation reduce brain-derived neurotrophic factor expression in first-episode psychosis: A pathway to smaller hippocampal volume. J. Clin. Psychiatry.

[69] Milan-Mattos J., Anibal F., Perseguini N., Minatel V., Rehder-Santos P., Castro C., Vasilceac F., Mattiello S., Faccioli L., Catai A. (2019). Effects of natural aging and gender on pro-inflammatory markers. Braz. J. Med. Biol. Res..

[70] Kent K., Charlton K., Roodenrys S., Batterham M., Potter J., Traynor V., Gilbert H., Morgan O., Richards R. (2017). Consumption of anthocyanin-rich cherry juice for 12 weeks improves memory and cognition in older adults with mild-to-moderate dementia. Eur. J. Nutr..

[71] Macready A., George T., Chong M.F., Alimbetov D.S., Jin Y., Vidal-Diez A., Spencer J.P.E., Kennedy O.B., Tuohy K.M., Minihane A.-M. (2014). Flavonoid-rich fruit and vegetables improve microvascular reactivity and inflammatory status in men at risk of cardiovascular disease—FLAVURS: A randomized controlled trial. Am. J. Clin. Nutr..

