# Supplementary material for: Neurocognitive Effects of Cocoa and Red-Berries Consumption in Healthy Adults
Source: Nutrients. 2021 Dec 21;14(1):1. doi: 10.3390/nu14010001 (PMC8746322; doi:10.3390/nu14010001)
Supplement: Supplementary file 1 [file nutrients-14-00001-s001.zip › Table s1.pdf]

Supplementary Table S1. Statistical results of the different neurocognitive by visit and treatment group.

	RB			C			RB-C			Total			P-value
	Baseline	12 w	p-value	Baseline	12 w	p-value	Baseline	12 w	p-value	Baseline	12 w	p-value	
Lissum	56.15 ± 8.10	58 ± 7.64	0.382	53.95 ± 7.84	58.15 ± 8.68	0.122	57.55 ± 89.96	54.11 ± 11.03	0.306	55.88 ± 8.10	56.77 ± 9.24	0.424	0.245
vlisARI	12.74 ± 1.82	12.37 ± 2.56	0.549	12.3 ± 2.22	12.15 ± 3.52	0.878	12.25 ± 2.46	11 ± 2.56	0.084	12.42 ± 2.16	11.84 ± 2.95	0.32	0.829
vlisARD	13.26 ± 1.44	12.74 ± 2.64	0.51	12.45 ± 2..48	12.05 ± 3.31	0.824	12.55 ± 2.62	12 ± 2.53	0.998	12.75 ± 2.24	12.26± 2.83	0.587	0.666
vRECTOT	42.16 ± 1.38	42.11 ± 2.83	0.818	42.00 ± 2.05	41.70 ± 1.92	0.677	42.50 ± 1.73	41.47 ± 2.24	0.041	42.22 ± 1.73	41.76 ± 1.88	0.189	0.495
VmvisuTOT	18.36 ± 5.45	19.21± 5.67	0.614	19.15 ± 4.80	19.20 ± 5.57	0.985	18.15 ± 5.61	19.42 ± 6.76	0.532	18.55 ± 5.22	19.27 ± 5.91	0.478	0.952
vmvisuRD	5.58 ± 2.38	6.32 ± 2.38	0.461	6.85 ± 2.13	6.75 ± 2.42	0.868	6.70 ± 2.34	6.63 ± 2.40	0.863	6.39 ± 2.31	6.57 ± 2.37	0.519	0.187
vLYNtot	19.42 ± 2.41	18.79 ± 2.93	0.337	18.90 ± 3.17	18.75 ± 3.22	0.844	18.6 ± 3.25	18.68 ± 2.64	0.861	18.97 ± 2.94	18.74 ± 2.89	0.742	0.567
vStroopP	100.37 ± 19.19	103.11 ± 19.02	0.717	101.40 ± 15.34	102.65 ± 15.09	0.681	94.45 ± 16.85	98.58 ± 22.92	0.205	98.71 ± 17.15	101.47 ± 18.98	0.337	0.618
vStroopC	100.37 ± 19.19	103.11 ± 19.02	0.472	101.40 ± 15.34	102.65 ± 15.09	0.984	94.45 ± 16.85	98.58 ± 22.92	0.177	98.71 ± 17.15	101.47 ± 18.98	0.27	0.297
vStroopPC	41.00 ± 11.51	40.26 ± 11.97	0.601	39.95 ± 14.42	40.75 ± 9.01	0.408	36.05 ± 11.13	38.58 ± 13.41	0.38	38.97 ± 12.42	39.88 ± 11.4	0.423	0.463
vTOLmov	95.84 ± 19.06	93.37 ± 17.92	0.481	92.45 ± 20.89	97.20 ± 15.37	0.313	95.4 ± 20.23	92.21 ± 19.56	0.658	94.54 ± 19.80	94.31 ± 24.91	0.892	0.809
vTOLstart	39.63 ± 18.83	45.84 ± 36.16	0.968	63.35 ± 32.57	47.6 ± 27.04	0.099	56.30 ± 40.27	39.26 ± 29.77	0.061	53.32 ± 32.99	44.29 ± 30.81	0.031	0.041
vTOLtot	255.37 ± 105.42	239 ± 98.88	0.601	306.65 ± 93.48	262 ± 72.81	0.052	313.9 ± 135.42	222.05 ± 75.56	0.008	292.59 ± 114.01	241.38 ± 83.27	0.007	0.157
vsymbol	49.74 ± 9.23	50.84 ± 11.18	0.569	49.7 ± 9.06	49.7 ± 12.22	0.087	47.6 ± 11.56	48 ± 10.91	0.077	49 ± 9.91	49.52 ± 11.32	0.012	0.89
vclaves	25.84 ± 8.14	27.48 ± 8.2	0.811	23.5 ± 5.96	27.68 ± 7.4	0.723	23.7 ± 7.69	27.26 ± 9.79	0.968	24.32 ± 7.26	27.26 ± 9.79	0.694	0.496
vcancel	27.37 ± 5.40	27.16 ± 6.47	0.887	25.85 ± 5.77	23.75 ± 4.55	0.337	24.6 ± 6.57	25.16 ± 7.03	0.448	25.92 ± 5.95	25.33 ± 6.14	0.722	0.396
vdigDtot	9.26 ± 1.4	8.68 ± 2.4	0.175	8.65 ± 1.78	9.8 ± 2.3	0.079	9.1 ± 1.94	8.74 ± 1.91	0.282	9 ± 1.72	9.09 ± 2.24	0.776	0.603
vdigItot	5.89 ± 2.62	5.84 ± 2.56	0.905	6.3 ± 1.8	6.65 ± 2.56	0.404	5.9 ± 2.75	5.79 ± 1.58	0.979	6.03 ± 2.39	6.1 ± 2.28	0.644	0.538

Data expressed as mean ± standard deviation (SD); significant difference at p-value < 0.05
